# Robust SAR Waveform Design for Extended Target in Spectrally Dense Environments

**DOI:** 10.3390/s25123670

**Published:** 2025-06-12

**Authors:** Rui Zhang, Fuwei Wu, Bing Gao, Ge Xu, Jianwei Wu, Jiawei Zhang

**Affiliations:** 1 Nanjing Research Institute of Electronics Technology, Nanjing 210039, China; zrui201@buaa.edu.cn (R.Z.); fuweiwu@cetc.com.cn (F.W.); binggao@cetc.com.cn (B.G.); gexu@cetc.com.cn (G.X.); 2 School of Information Science and Engineering, Yanshan University, Qinhuangdao 066004, China; wujianwei@stumail.ysu.edu.cn

**Keywords:** SAR, SCR, extended targets, robust waveform, spectral constraint

## Abstract

To enhance the signature of an extended target in a SAR image, a robust waveform design method is presented for spectrally dense environments. First, the problem is formulated by maximizing the worst-case signal-to-clutter ratio (SCR) over the uncertainty set of statistics for both target and background scattering characteristics, subject to energy, similarity, and spectrum constraints. Second, the closed-form solutions for the uncertain statistics are derived. The problem of maximizing worst-case SCR is boiled down to a nonconvex fractional quadratically constrained quadratic problem (QCQP). Resorting to the Dinkelbach’s algorithm and Lagrange duality, the QCQP is split into a series of solvable semidefinite programming problems. A convergence analysis is conducted, where a sufficient condition for global convergence is derived. Finally, numerical examples are presented to demonstrate the performance of the proposed scheme.

## 1. Introduction

Synthetic aperture radar (SAR) is an active microwave imaging technique and can provide high-resolution images independent from daylight and weather [1,2]. The optimization of transmit waveforms is a longstanding issue, requiring high resolution and sidelobe suppression under point target assumptions. Based on the spectrum shape, they can be mainly divided into three categories: linear frequency modulation (LFM), stepping frequency (SF), and nonlinear frequency modulation (NLFM).

The LFM waveform with the rectangular spectrum shape has been widely utilized in practical SAR systems. With a large time-bandwidth product, simple generation, and good Doppler tolerance, it achieves desired point-like target detection performance, as demonstrated by a good shape of correlation function (CF) with a narrow main lobe and sidelobe level up to −13.3 dB [3]. Also, to pursue high resolution for a point target, the SF waveform usually has a wide band width, whose frequency changes in the way of off-walk. The main idea is to divide the large-bandwidth signal into multiple transmissions, thereby reducing the instantaneous bandwidth of the system [4,5].

The NLFM waveform shapes the power spectrum such that the CF exhibits reduced sidelobes. No additional filtering is required, so that the signal-to-noise ratio (SNR) degradation can be avoided [6]. Jin et al. [7] reported a novel waveform optimization framework where an advanced NLFM waveform with lower sidelobes and a higher SNR of 1.29 dB was constructed. In [8], the authors proposed a scheme with reciprocating NLFM for the quasi-orthogonal waveform design, and the sidelobe peak level of the optimized waveform was reduced by 5.5–9.5 dB.

The above SAR waveform designs are oriented to a desired CF with a narrower mainlobe and lower sidelobes. Although these properties are basic conditions for SAR to provide an image with more details, the pursuit of point-like target detection performance does not indicate a desired imaging quality for extended targets. The returns of extended targets are not the scaled and attenuated version of the transmit waveform, but its convolution with the target impulse response (TIR) [9,10,11,12]. Moreover, with continuing advances in resolution, targets can occupy multiple resolution cells of the sensor, giving rise to more than one measurement per time step. The point target assumption no longer holds in such scenarios [13]. Thus, specifically designed waveforms for extended targets are needed [14]. Moreover, as SAR usually desires a wide bandwidth to guarantee high resolution, the coexistence of wideband systems with other radio frequency transmitters becomes a serious issue. For these reasons, it is of great importance to study the waveform design problem in spectrally dense environments.

Although the work for SAR waveform designs is rarely reported in the literature for extended targets, there have been a multitude of studies for other radar systems, which can be divided into three categories based on the optimization criterion: signal power [15,16], information theory [17,18], and ambiguity function [19,20]. Among them, the power-based criterion is widely used in waveform optimization, as it facilitates signal modeling and enhancement of weak target scattering.

Based on the scattering model, the power-based methods can be classified into two groups: deterministic and stochastic extended targets, considering the TIR as deterministic and random processes, respectively.

- Deterministic target model

In [21], a joint transmit–receive optimization approach was proposed to facilitate the optimal detection of a deterministic target, by maximizing the output signal-to-interference plus noise ratio (SINR). This approach has been further applied for target identification through waveform optimization [22,23]. Both the SNR and mutual information criteria were employed in [24] for waveform design, to improve the performance of a closed-loop radar system for known TIR target recognition purposes. Furthermore, the waveform optimization framework provided in [21] was generalized to benefit multiple-input multiple-output (MIMO) radar performance [25]. This technique is not guaranteed to converge on an optimal solution, while the method in [26] sequentially improves the SINR for a known TIR in signal-dependent interference, with a proof for convergence. Similarly, an iterative algorithm maximizing the SINR as a waveform design figure of merit was developed to improve the detection performance of a deterministic target in [27], and the methodology was further applied to [21], with significantly improved performance compared to the chirp signal.

- Stochastic target model

Considering that TIR is sensitive to the line of sight, it is relaxed to an uncertainty set or a random vector, leading to a stochastic target model that is more general than the deterministic one [12,26,28,29,30,31]. A novel iterative algorithm was proposed to optimize the waveform and receiving filter such that the detection performance could be maximized, under the case with knowledge of only the statistics or the uncertainty set of the TIR [26]. A robust design method to jointly optimize the radar transmit code and receive filter was proposed in [28] with two different uncertainty sets by exploiting the SINR at the receiver end. Based on the stochastic model of TIR, Yao et al. developed robust design methods to optimize the detection of extended targets in the presence of signal-dependent interference [29], where the lowest SINR was considered as the performance measure of the system. The design method was extended in [30], where two iterative optimization schemes were developed to optimize the average and worst-case SINR. With the partially known TIR falling into an uncertain set, a novel waveform design method was developed in [31], where SINR was formed as the objective function, subject to the peak-to-average power ratio constraint. Xu et al. [12] further generalized the work in [31] to the signal dependent interference environment, and the designed waveform–filter pair was shown to be more robust.

Moreover, the extended-target waveform design with a spectral constraint has attracted a lot of interest in recent years, given the emergence of new wideband and ultra-wideband radar systems with a strong demand for spectra.

According to the analysis in [32], an optimization design method for phase-only waveform synthesis involving spectrally dense environments is proposed assuming that SINR is the figure of merit. Unlike existing approaches which design the waveform for a single target via output SINR maximization [33], a spectrally compatible waveform design for MIMO radar in the presence of multiple targets and signal-dependent interference was studied in [34]. Similarly, Aubry et al. considered the design of radar waveforms in a spectrally crowded case [35], and the waveform performance was studied in terms of a trade-off among the achievable SINR, spectral shape, and autocorrelation function. In [36], a new technique for constant modulus waveform synthesis specifically designed for spectrally dense environments was proposed, using SINR as the performance metric. Furthermore, to improve multiple targets’ detectability under spectral constraints, the joint design of a MIMO radar transmit waveform and receive filter was studied in [37]. In [38], the distribution of output SINR was derived, and a novel waveform design method was proposed to improve detection performance. A robust joint design of the radar code and receive filter bank guaranteeing spectral compatibility was studied in [39], where the average SINR was considered as the performance measure. In [40], Ding et al. introduced the multi-group optimization problem of MIMO radar by optimizing the output signal-to-clutter-noise ratio (SCNR) with the spectrum constraint via a cyclic algorithm.

The incorporation of robustness usually results in a highly complex max–min fractional optimization problem with nonconvex quadratic constraints, making conventional optimization approaches inapplicable or inefficient. In this paper, a customized algorithm is developed to tackle the fractional non-convex optimization problem arising from robust waveform design, combining a closed-form solution for minimization subproblems with iterative semidefinite programming (SDP) solving based on Lagrange duality and the Schur complement. The problem is formulated as maximizing the worst-case SCR over the uncertain set of statistics, subject to energy, similarity, and spectral constraints. To solve the complicated problem with coupled max–min operators, a customized procedure with polynomial-time complexity is proposed. A closed-form solution for the statistics is derived, such that the problem is boiled down to a nonconvex fractional quadratically constrained quadratic problem (QCQP). The Dinkelbach’s algorithm is applied to decouple the fractional objective function into a quadratic function. Followed by the Lagrange duality, the QCQP is tackled by solving a series of SDP problems. The convergence is guaranteed by the solving method, and a sufficient condition for global convergence is also provided. As demonstrated by computer simulations, the robust SAR waveform ensures the SCR of extended targets to be sufficiently high for any statistics in the uncertainty class and also allows other radiators to coexist in spectrally crowded environments.

The rest of this paper is organized as follows. Section 2 provides the signal model and formulates the constrained optimization problem for robust SAR waveform design. In Section 3, the solving procedure is customized, followed by the convergence and complexity analysis. Numerical results are presented in Section 4, and conclusions are drawn in Section 5.

Notation: Throughout this article, matrices are denoted by bold uppercase letters and vectors by bold lowercase letters. C and $C^{N}$ represent the set of complex and *N*-dimensional complex column vectors, respectively. The N×N complex Hermitian space is denoted by $H^{N}$. ℜ(A) denotes the range space of the matrix. $A^{\left(1\right)}$ is the pseudo-inverse of A, and $A^{-1}$ represents the inverse of A. 1 and I are the all-one and identity matrices. The superscript ${\left(\cdot \right)}^{H}$ represents the Hermitian transpose. ∥·∥ and ${∥\cdot ∥}_{F}$ denote the Euclidean norm and the Frobenius norm. tr[·] and E[·] represent the trace of a matrix and expectation operation, respectively. ∇ and L represent the differential operator and Lagrange function, respectively. The notation A⪰B and A≻B means that A−B is positive semidefinite and positive definite, separately. Finally, for any optimization problem P, V(P) and S(P) represent its optimal value and the set of optimal solutions, respectively.

## 2. Problem Formation

Without loss of generality, the extended target TIR G(t) is modeled as a general stationary random process, and it interacts with the transmit waveform x(t) to generate a scattering signal. Corrupted by background scattering B(t) and noise N(t), the signal received from G(t) is [28]

(1) Y(t)=G(t)⊗x(t)+B(t)⊗x(t)+N(t).

where ⊗ is the convolution operator. In discrete form, by using Toeplitz matrix construction to implement the convolution operator, we have

(2) y=Gx+Bx+n,

where G and B are Toeplitz matrices formed by discretizing G(t) and B(t), respectively, and $x,\;y,\;n\in C^{N}$ are discrete samples of x(t), Y(t), and N(t), respectively. Suppose that G(t), B(t), and N(t) are statistically independent, and then the SCR of a SAR range line is

(3) $$\mathrm{SCR}=\frac{E[x^{H}G^{H}\mathrm{Gx}]}{E[x^{H}B^{H}\mathrm{Bx}]}=\frac{x^{H}R_{G}x}{x^{H}R_{B}x},$$

where $R_{G}=E\left[G^{H}G\right]$ and $R_{B}=E\left[B^{H}B\right]$ are correlation matrices involving the second-order statistics of G(t) and B(t), respectively. In our previous work [18], a joint design method for SAR waveform and filters was proposed based on maximizing MI, which achieved better detection performance by utilizing greater freedom resulting from range and azimuth filters. However, it is more sensitive to the accuracy of prior information, and its performance degrades with prior mismatch. To this end, we focuses on a robust waveform design under an uncertainty set of statistics for both target and background scattering characteristics, which ensures a high signal-to-clutter ratio (SCR) for any priori information within the uncertainty set. Due to the estimation error of statistics, the actual $R_{G}$ and $R_{B}$ are not known exactly, so they lie in an uncertain region, i.e., [26,41,42]

(4) $${∥R_{G}-{\tilde{R}}_{G}∥}_{F}^{2}\leq \varepsilon_{G}\;\mathrm{and}\;{∥R_{B}-{\tilde{R}}_{B}∥}_{F}^{2}\leq \varepsilon_{B},$$

where ${\tilde{R}}_{G}$ and ${\tilde{R}}_{B}$ are the nominal correlation matrices, and $\varepsilon_{G}$ and $\varepsilon_{B}$ are parameters used to control the size of the uncertain set. The worst-case SCR regarding uncertain $R_{G}$ and $R_{B}$ is given by

$$P_{0}\left\{\begin{array}{c} \min_{R_{G},R_{B}}\frac{x^{H}R_{G}x}{x^{H}R_{B}x}\\ \mathrm{s.t.}\left\{\begin{array}{c} {∥R_{G}-{\tilde{R}}_{G}∥}_{F}^{2}\leq \varepsilon_{G}\\ {∥R_{B}-{\tilde{R}}_{B}∥}_{F}^{2}\leq \varepsilon_{B} \end{array}\right. \end{array}\right.$$

To prevent poor resolution, significant modulus fluctuation, or even unrealizability, appropriate constraints must be introduced into the optimization process. Firstly, the energy of a transmit waveform is limited, i.e., $x^{H}x\leq E_{x}$ with maximum allowable energy $E_{x}$. With preferred wideband and low sidelobe properties, a similarity constraint with a chirp signal $x_{0}$ is introduced as

(5) $${∥x-x_{0}∥}_{2}^{2}\leq \varepsilon ,$$

where ε is the similarity parameter. Moreover, wideband emissions often overlap with other radio frequency systems, necessitating spectral constraints to ensure coexistence. Concerning licensed systems coexisting with the radar of interest, it is supposed that each of them is operating over a frequency band $\Omega_{q}=\left[f_{1}^{q},f_{2}^{q}\right],\;q=1,\ldots ,Q$, with $f_{1}^{q}$ and $f_{2}^{q}$ denoting the lower and upper normalized frequencies for the *q*-th radiator, respectively. The spectral constraint is then expressed as

(6) $$x^{H}R_{I}x\leq E_{I}$$

with $R_{I}=\sum_{q=1}^{Q}\gamma_{q}\Omega_{q}$, where the (m,l)th entry of $\Omega_{q}$ is given by

(7) $$\Omega_{q}\left(m,l\right)=\left\{\begin{array}{c} \frac{\exp\{j2\pi f_{2}^{q}\left(m-l\right)\}-\exp\{j2\pi f_{1}^{q}\left(m-l\right)\}}{j2\pi (m-l)},\;m\neq l\\ f_{2}^{q}-f_{1}^{q},\;m=l \end{array}\right.$$

for $\left(m,l\right)\in {\left\{1,\ldots N\right\}}^{2}$ [35,39]. Note that $\gamma_{q}\geq 0$ are the coefficients given to different systems, and $E_{I}$ denotes the maximum energy of allowed interference tolerated by other radiators.

In practical engineering applications, the estimation of the statistical characteristics of target scattering inevitably involves certain errors. When there is a mismatch between the prior information and the actual application scenario, it often results in performance degradation. To enhance system robustness, we adopt the criterion of maximizing the worst-case SCR, which improves tolerance to errors in prior information and ensures more reliable performance under uncertain sets. Robust waveform design aims at determining x so as to maximize the SCR, while the worst-case cost is minimized under all possible $R_{G}$ and $R_{B}$. Collecting all constraints, the robust design problem of x is finally formed as

$$P_{1}\left\{\begin{array}{c} \max_{x}\;\min_{R_{G},R_{B}}\;\frac{x^{H}R_{G}x}{x^{H}R_{B}x}\\ \mathrm{s.t.}\left\{\begin{array}{c} x^{H}x\leq E_{x}\\ {∥x-x_{0}∥}_{2}^{2}\leq \varepsilon \\ x^{H}R_{I}x\leq E_{I}\\ {∥R_{G}-{\tilde{R}}_{G}∥}_{F}^{2}\leq \varepsilon_{G}\\ {∥R_{B}-{\tilde{R}}_{B}∥}_{F}^{2}\leq \varepsilon_{B} \end{array}\right. \end{array}\right.$$

## 3. Solving the Problem

### 3.1. Algometric Procedure

Due to the complicated max–min form coupled with a quadratic fractional objective function, it is not easy to find a desired solution through the existing optimization algorithms for $P_{1}$. In this section, we devise a customized method to enhance the worst-case SCR over the uncertain region in presence of multiple practical constraints. As the first step toward this goal, a closed-form solution pair for $R_{G}$ and $R_{B}$ is obtained by transforming $P_{1}$. Reconsider $P_{0}$, and we introduce two auxiliary variables $\Delta R_{G},\;\Delta R_{B}\in H^{N}$ with $\Delta R_{G}=R_{G}-{\tilde{R}}_{G}$ and $\Delta R_{B}=R_{B}-{\tilde{R}}_{B}$, so that $P_{0}$ is transformed into

$$P_{2}\left\{\begin{array}{c} \min_{\Delta R_{G},\Delta R_{B}}\frac{x^{H}\left(\Delta R_{G}+{\tilde{R}}_{G}\right)x}{x^{H}\left(\Delta R_{B}+{\tilde{R}}_{B}\right)x}\\ \mathrm{s.t.}\left\{\begin{array}{c} {∥\Delta R_{G}∥}_{F}^{2}\leq \varepsilon_{G}\\ {∥\Delta R_{B}∥}_{F}^{2}\leq \varepsilon_{B} \end{array}\right. \end{array}\right.$$

As can be seen, when the numerator reaches its minimum value, and the maximum value is reached for the denominator, the objective function obtains its optimal solution. Thus, ignoring the terms unrelated to the decision variables, $P_{2}$ can be modified as

$$P_{3}\left\{\begin{array}{c} \frac{\min_{\Delta R_{G}}\;x^{H}\Delta R_{G}x}{\max_{\Delta R_{B}}\;x^{H}\Delta R_{B}x}\\ \mathrm{s.t.}\left\{\begin{array}{c} {∥\Delta R_{G}∥}_{F}^{2}\leq \varepsilon_{G}^{2}\\ {∥\Delta R_{B}∥}_{F}^{2}\leq \varepsilon_{B}^{2} \end{array}\right. \end{array}\right.$$

Since there is no coupling between $\Delta R_{G}$ and $\Delta R_{B}$ in either the objective function or the constraints, $P_{3}$ can be split into two independent subproblems, i.e.,

$$P_{4}\left\{\begin{array}{c} \min_{\Delta R_{G}}\;x^{H}\Delta R_{G}x\\ \mathrm{s.t.}{∥\Delta R_{G}∥}_{F}^{2}\leq \varepsilon_{G}^{2} \end{array}\right.\mathrm{and}\;P_{5}\left\{\begin{array}{c} \max_{\Delta R_{B}}\;x^{H}\Delta R_{B}x\\ \mathrm{s.t.}{∥\Delta R_{B}∥}_{F}^{2}\leq \varepsilon_{B}^{2} \end{array}\right.$$

As linear objective functions of both problems with only one quadratic constraint, they can be changed to linear programming (LP) problems:

$$P_{6}\left\{\begin{array}{c} \min_{\Delta R_{G}}\;x^{H}\Delta R_{G}x\\ \mathrm{s.t.}\;-\varepsilon_{G}1⪯\Delta R_{G}⪯\varepsilon_{G}1 \end{array}\right.\mathrm{and}\;P_{7}\left\{\begin{array}{c} \max_{\Delta R_{B}}\;x^{H}\Delta R_{B}x\\ \mathrm{s.t.}\;-\varepsilon_{B}1⪯\Delta R_{B}⪯\varepsilon_{B}1 \end{array}\right.$$

where their solutions are given by $\Delta R_{G}^{†}=-\varepsilon_{G}1$ and $\Delta R_{B}^{†}=\varepsilon_{B}1$, respectively. Therefore, it further leads to the optimal solutions $R_{G}^{†}={\tilde{R}}_{G}-\varepsilon_{G}1$ and $R_{B}^{†}={\tilde{R}}_{B}+\varepsilon_{B}1$ for $R_{G}$ and $R_{B}$, respectively. Substituting them into (2), $P_{1}$ is boiled down to

$$P_{8}\left\{\begin{array}{c} \max_{x}\;\frac{x^{H}R_{G}^{†}x}{x^{H}R_{B}^{†}x}\\ \mathrm{s.t.}\left\{\begin{array}{c} x^{H}x\leq E_{x}\\ {∥x-x_{0}∥}_{2}^{2}\leq \varepsilon \\ x^{H}R_{I}x\leq E_{I} \end{array}\right. \end{array}\right.$$

Herein, the difficulty in solving $P_{8}$ lies in $f\left(x\right)=x^{H}R_{G}^{†}x/x^{H}R_{B}^{†}x\;$, but its numerator and denominator can be decoupled to form a quadratic function, resorting to the Dinkelbach’s algorithm [43]. To this end, an iteration loop with index *k* is performed, where $P_{8}$ is split into a series of subproblems. At the *k*-th step, we have

(8) $$P_{9}\left\{\begin{array}{c} x^{\left(k\right)}=\arg\max_{x}\;x^{H}R_{G}^{†}x-f\left(x^{\left(k-1\right)}\right)x^{H}R_{B}^{†}x\\ \mathrm{s.t.}\left\{\begin{array}{c} x^{H}x\leq E_{x}\\ {∥x-x_{0}∥}_{2}^{2}\leq \varepsilon \\ x^{H}R_{I}x\leq E_{I} \end{array}\right. \end{array}\right.$$

By iterating $P_{9}$, a monotonically increasing sequence ${\left\{f\left(x^{\left(k\right)}\right)\right\}}_{k=1}^{\infty }$ is obtained [43]. Meanwhile, if $P_{9}$ can be solved globally in each iteration, global convergence is guaranteed for $P_{8}$, which is proved by detailed derivations in Appendix A.1. Let $h\left(x\right)=x^{H}R_{G}^{†}x-f\left(x^{\left(k\right)}\right)x^{H}R_{B}^{†}x$, and it is either convex or concave. These two cases are treated separately. Firstly, if $R_{G}^{†}⪯f\left(x^{\left(k\right)}\right)R_{B}^{†}$, then h(x) is concave, and $P_{9}$ is a convex QCQP. Using the CVX tool, its optimal solution can be found with polynomial complexity.

On the contrary, if $R_{G}^{†}⪰f\left(x^{\left(k\right)}\right)R_{B}^{†}$, h(x) is convex, and $P_{9}$ is a nonconvex QCQP, the solving procedure is provided as follows. For the sake of clarity, $P_{9}$ is reformulated into a general form, i.e.,

$$P_{10}\left\{\begin{array}{c} \min_{x}\;x^{H}A_{0}x+b_{0}^{H}x+x^{H}b_{0}+c_{0}\\ \mathrm{s.t.}\;x^{H}A_{i}x+b_{i}^{H}x+x^{H}b_{i}+c_{i}\leq 0,\;i=1,2,3 \end{array}\right.$$

where $A_{0}=f\left(x^{\left(k\right)}\right)R_{B}^{†}-R_{G}^{†}$, $A_{1}=A_{2}=I$, $A_{3}=R_{I}$, $b_{0}=b_{1}=b_{3}=0$, $b_{2}=-c$, $c_{0}=0$, $c_{1}=-E_{x}$, $c_{2}=E_{0}-\varepsilon$ with $E_{0}=x_{0}^{H}x_{0}$, and $c_{3}=-E_{I}$. The Lagrange is

(9) $$\begin{array}{c} L\left(x,\lambda \right)=x^{H}A_{0}x+b_{0}^{H}x+x^{H}b_{0}+\sum_{i=1}^{3}\lambda_{i}\left(x^{H}A_{i}x+b_{i}^{H}x+x^{H}b_{i}+c_{i}\right) \end{array}$$

with multipliers $\lambda_{1}$, $\lambda_{2}$, and $\lambda_{3}$. The duality function is $g\left(\lambda \right)=\underset{x}{\inf}\;L\left(x,\lambda \right)$. It is an unconstrained problem for x, and we can find the minimizing x from $\nabla_{x}L\left(x,\lambda \right)=0$, while

(10) $$g\left(\lambda \right)=\left\{\begin{array}{c} c\left(\lambda \right)-b{\left(\lambda \right)}^{H}A{\left(\lambda \right)}^{\left(1\right)}b\left(\lambda \right),\;A\left(\lambda \right)\underline{≻}0,\;b\left(\lambda \right)\in ℜ\left[A\left(\lambda \right)\right]\\ -\infty ,\;\mathrm{otherwise} \end{array}\right.$$

where $A\left(\lambda \right)=A_{0}+\sum_{i=1}^{3}\lambda_{i}A_{i}$, $b\left(\lambda \right)=b_{0}+\sum_{i=1}^{3}\lambda_{i}b_{i}$, $c\left(\lambda \right)=\sum_{i=1}^{3}\lambda_{i}c_{i}$, and $A{\left(\lambda \right)}^{\left(1\right)}$ is the pseudo-inverse of A(λ). The Lagrange dual problem is

$$P_{11}\left\{\begin{array}{c} \max_{\lambda_{i}}\;g\left(\lambda \right)\\ \mathrm{s.t.}\left\{\begin{array}{c} A\left(\lambda \right)\underline{≻}0\\ b(\lambda )\in ℜ[A(\lambda )]\\ \lambda_{i}\geq 0,\;i=1,2,3 \end{array}\right. \end{array}\right.$$

It is still a nonconvex problem, while its epigraph form is

$$P_{12}\left\{\begin{array}{c} \max_{\lambda_{i},\alpha }\;\alpha \\ \mathrm{s.t.}\left\{\begin{array}{c} g(\lambda )\geq \alpha \\ A\left(\lambda \right)\underline{≻}0\\ b(\lambda )\in ℜ[A(\lambda )]\\ \lambda_{i}\geq 0,\;i=1,2,3 \end{array}\right. \end{array}\right.$$

Based on the Schur complementary theorem [44], an equivalent SDP problem is

$$P_{13}\left\{\begin{array}{c} \max_{\lambda_{i},\alpha }\;\alpha \\ \mathrm{s.t.}\left\{\begin{array}{c} \left[\begin{array}{cc} A(\lambda ) & b(\lambda )\\ b{\left(\lambda \right)}^{H} & c(\lambda )-\alpha \end{array}\right]\underline{≻}0\\ \lambda_{i}\geq 0,\;i=1,2,3 \end{array}\right. \end{array}\right.$$

Based on the above procedure, $P_{9}$ is finally transformed into a convex problem $P_{13}$. Suppose $\lambda_{1}^{†}$, $\lambda_{2}^{†}$, and $\lambda_{3}^{†}$ are optimal solutions of $P_{13}$, then $x^{\left(k\right)}=-A{\left(\lambda^{†}\right)}^{\left(1\right)}b\left(\lambda^{†}\right)$. In conclusion, $P_{8}$ is tackled by solving $P_{13}$, until the iteration loop is stopped. One reasonable stopping condition is that

$$f\left(x^{\left(k\right)}\right)-f\left(x^{\left(k-1\right)}\right)\leq e$$

where *e* represents the minimum variation of the objective function. The overall algorithmic procedure is summarized as Algorithm 1:

**Algorithm 1:** Solving procedure for $P_{1}$ **Input** decision variables x, $R_{G}$, and $R_{B}$. **Initialize** $x^{\left(0\right)}$, $E_{x}$, ε, $E_{I}$, $\varepsilon_{G}$, $\varepsilon_{B}$, *e* **Form** $P_{1}$ with $x_{0}$, $R_{I}$, ${\tilde{R}}_{G}$ and ${\tilde{R}}_{B}$ **Find** the closed-form solutions for $R_{G}$ and $R_{B}$ **Form** $P_{8}$ with $R_{G}^{†}$ and $R_{B}^{†}$

 k:=1

 
**Repeat**
          
Construct the Lagrange duality          
Form $P_{13}$ and solve it. **until** stopping criterion is satisfied. **output** $x^{†}=x^{\left(K\right)}$ with maximum iteration step *K*.

Here, the initial point $x^{\left(0\right)}$ is a feasible point satisfying all constraints, which can be determined by the following problem:

$$P_{14}\left\{\begin{array}{c} \min_{x^{\left(0\right)}}\;x^{(0)H}R_{I}x^{\left(0\right)}\\ \mathrm{s.t.}\left\{\begin{array}{c} x^{(0)H}x^{\left(0\right)}\leq E_{x}\\ {∥x^{\left(0\right)}-x_{0}∥}_{2}^{2}\leq \varepsilon \end{array}\right. \end{array}\right.$$

### 3.2. Convergence and Complexity Analysis

Reconsidering the original problem $P_{1}$, its convergence verification involves two parts, which are minimization and maximization operators. The first one achieves closed-form solutions for both $R_{G}$ and $R_{B}$, leading to a global convergence. The convergence of the maximization operator is determined by the sequence ${\left\{f\left(x^{\left(k\right)}\right)\right\}}_{k=1}^{\infty }$. Since f(x) is upper bounded, ${\left\{f\left(x^{\left(k\right)}\right)\right\}}_{k=1}^{\infty }$ finally converges to a limit point. Its global convergence depends on whether $P_{9}$ admits a global solution. As mentioned before, $P_{9}$ is transformed into $P_{13}$, resorting to the Lagrange duality. There always exists the weak duality inequality $V\left(P_{13}\right)\leq V\left(P_{9}\right)$. If the duality gap is zero, the strong duality holds with $V\left(P_{13}\right)=V\left(P_{9}\right)$, so that the optimal solution of $P_{9}$ is obtained by solving $P_{13}$. As the strong duality does not hold in general, a sufficient condition for the strong duality is derived. To this end, we introduce the following lemma, and its proof is provided in Appendix A.2.

**Lemma 1.**  *With $\alpha^{†}$, $\lambda_{i}^{†}\in S\left(P_{13}\right)$, i=1,2,3, if $A(\lambda^{†})≻0$, then $x^{†}=-A{\left(\lambda^{†}\right)}^{-1}b\left(\lambda^{†}\right)$ is optimal to $P_{9}$ with zero duality gap.*

Therefore, after completing the iteration, if the obtained $\alpha^{†}$, $\lambda_{i}^{†}\in S\left(P_{13}\right)$ allows the condition of Lemma 1 to be met, then the zero duality gap is achieved to guarantee global convergence of $P_{1}$. On the contrary, if Lemma 1 does not hold, a global convergence may not always be guaranteed. If this undesired case happens, $P_{9}$ can also be solved globally by an alternative method, but it causes more computational burden and more memory. Resorting to [45], $P_{9}$ is firstly written as an semidefinite relaxation (SDR):

(11) $$P_{\mathrm{SDR}}\left\{\begin{array}{c} X^{\left(k\right)}=\arg\max_{X}\;\mathrm{tr}\left\{[R_{G}^{†}-f\left(x^{\left(k-1\right)}\right)R_{B}^{†}]X\right\}\\ \mathrm{s.t.}\left\{\begin{array}{c} \mathrm{tr}\left[\mathrm{IX}\right]⪯E_{x}\\ \mathrm{tr}\left[\left(x_{0}x_{0}^{H}\right)X\right]⪰\varepsilon^{\prime }\\ \mathrm{tr}\left[R_{I}X\right]⪯E_{I}\\ X⪰0 \end{array}\right. \end{array}\right.$$

where rank-one constraint $X=xx^{H}$ is relaxed, and $\varepsilon^{\prime }={\left[\left(2x_{0}^{H}x_{0}-\varepsilon \right)/2\;\right]}^{2}$. Since $X^{\left(k\right)}$ solves $P_{\mathrm{SDR}}$, we always have

(12) $$\begin{array}{c} \mathrm{tr}\left\{\left[R_{G}^{†}-f\left(x^{\left(k-1\right)}\right)R_{B}^{†}\right]X^{\left(k\right)}\right\}\geq \mathrm{tr}\left\{\left[R_{G}^{†}-f\left(x^{\left(k-1\right)}\right)R_{B}^{†}\right]xx^{H}\right\} \end{array}$$

where $X=xx^{H}$ is a feasible solution for $P_{\mathrm{SDR}}$. Followed by an efficient rank-one decomposition, one can find in polynomial time, a desired solution $X^{\left(k\right)}=x^{\left(k\right)}x^{(k)H}$ such that [45,46]

(13) $$\begin{array}{c} \mathrm{tr}\left\{\left[R_{G}^{†}-f\left(x^{\left(k-1\right)}\right)R_{B}^{†}\right]X^{\left(k\right)}\right\}=x^{(k)H}\left[R_{G}^{†}-f\left(x^{\left(k-1\right)}\right)R_{B}^{†}\right]x^{\left(k\right)} \end{array}$$

Thus, it is guaranteed that $P_{9}$ converges to a globally optimal point, which further indicates a global convergence for $P_{1}$, regardless of whether Lemma 1 holds or not.

The complexity lies in iterating $P_{13}$, whose worst-case complexity is of $O(\max{\left\{N,N^{\prime }\right\}}^{4}N^{0.5})$ where $N^{\prime }$ denotes the number of constraints [47]. Meanwhile, if Lemma 1 is violated, an extra complexity of $O(N^{3})$ is caused by the rank-one decomposition [45].

## 4. Numerical Examples

This section provides several numerical examples firstly to verify the theoretical derivations, including convergence of algorithmic procedure, robustness of uncertain statistics, and effectiveness of constraints. Then, the proposed scheme is applied to SAR, where performance evaluation is carried out.

### 4.1. Theoretical Derivation Verification

#### 4.1.1. Convergence Validation

**Figure 1:**
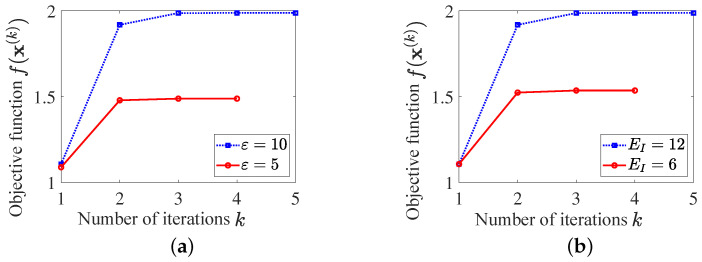
Objective function value with different feasible regions: (**a**) with different ε; (**b**) with different $E_{I}$.

Set N=120, $e=10^{-4}$, $E_{x}=E_{c}=N$, and $\varepsilon_{G}^{2}=\varepsilon_{B}^{2}=10$, and the reference waveform $x_{0}$ is a chirp signal. Considering different feasible regions resulting from varied similarity parameter ε and spectral parameter $E_{I}$, Algorithm 1 is performed, and the objective function $f(x^{\left(k\right)})$ versus iteration step *k* is presented in Figure 1.

As can be seen, the convergence of Algorithm 1 is guaranteed by the monotonically increasing objective function sequence, which converges rapidly to a desired point after 4 iterations. It means that the customized algorithm is capable of solving $P_{1}$. As expected, no matter which one of ε and $E_{I}$ increases, a larger feasible region is obtained, which further leads to a higher objective function value.

#### 4.1.2. Robustness Property

**Figure 2:**
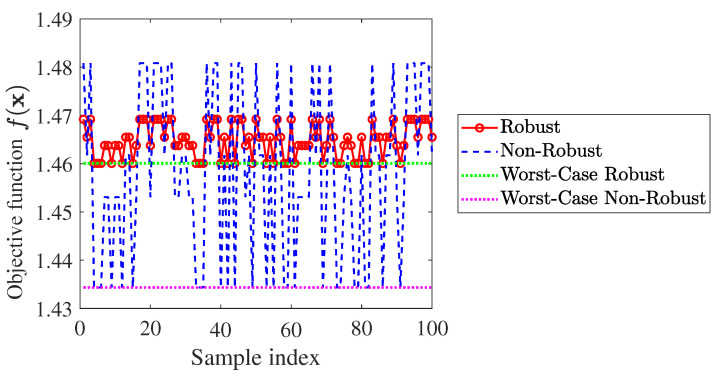
Achieved f(x) versus random samples of $R_{G}$ and $R_{B}$.

To evaluate the robustness of the proposed waveform, we compute the achievable objective function under various randomly generated $R_{G}$ and $R_{B}$. A total of 100 groups of samples from the uncertain set are randomly picked up to calculate f(x) provided by the robust waveform. For the sake of comparison, the non-robust design is considered as a counterpart, where no uncertainty of $R_{G}$ and $R_{B}$ is assumed at the design stage. Meanwhile, the worst-case f(x) for both robust and non-robust cases are also plotted in Figure 2.

The simulation results suggest that the proposed scheme exhibits a desired behavior for the fluctuated statistics $R_{G}$ and $R_{B}$, as robustness is achieved by maximizing the worst-case f(x) over the uncertainty set. As expected, the robust waveform always maintains a steadily high value of f(x), regarding the varied $R_{G}$ and $R_{B}$. Meanwhile, the worst-case f(x) provided by the robust waveform is significantly higher than that of the non-robust one, which further indicates that the robustness is guaranteed well.

#### 4.1.3. Optimality Condition

**Figure 3:**
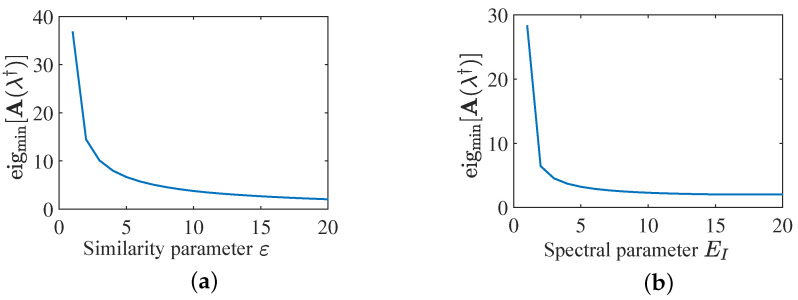
The smallest eigenvalue of $A(\lambda^{†})$ versus different feasible regions: (**a**) varied similarity parameter ε; (**b**) varied spectral parameter $E_{I}$.

As mentioned before, whether $P_{1}$ can achieve a global convergence relies on the convergence of $P_{9}$. If $P_{9}$ obtains an optimal solution, then $P_{1}$ can be solved globally. Based on Lemma 1, it provides a sufficient condition $A(\lambda^{†})≻0$ for a zero duality gap between $P_{9}$ and its dual problem. As $A(\lambda^{†})≻0$ is equivalent to ${\mathrm{eig}}_{\min}\left[A\left(\lambda^{†}\right)\right]>0$, the smallest eigenvalue of $A(\lambda^{†})$ is presented versus different feasible regions in Figure 3.

**Figure 4:**
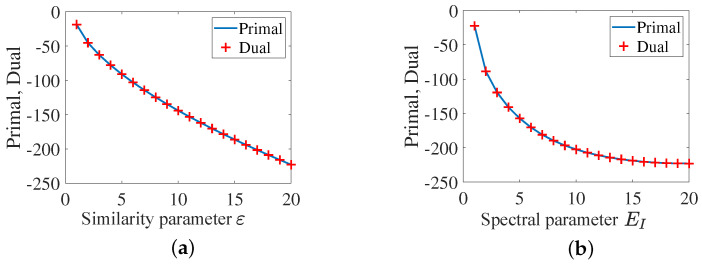
The objective functions for both primal and dual problems: (**a**) with different ε; (**b**) with different $E_{I}$.

Figure 3 indicates that ${\mathrm{eig}}_{\min}\left[A\left(\lambda^{†}\right)\right]>0$ is satisfied over all considered feasible regions, so that the optimality condition is met. To exhibit the duality gap between $P_{9}$ and $P_{11}$, we also plot the objective functions for both primal and dual problems, as shown in Figure 4.

It is clear that the primal problem and the dual one have the same objective function value, which demonstrates zero duality gap. Therefore, a global convergence of $P_{9}$ is achieved, such that $P_{1}$ is guaranteed to converge on a global point.

#### 4.1.4. Spectral Compatibility

**Figure 5:**
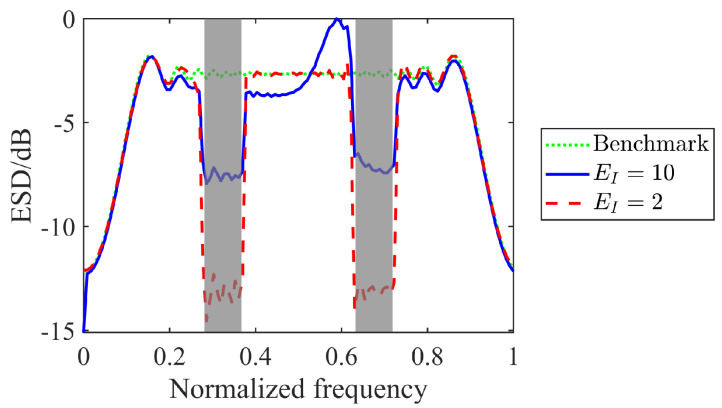
ESDs of optimized waveforms versus varied $E_{I}$, where the stopbands are shaded in light gray.

To validate the spectral constraint, assume two wireless radiators operating over normalized frequency bands $\Omega_{1}=\left[0.27,0.37\right]$ and $\Omega_{2}=\left[0.62,0.72\right]$ calculated by dividing their actual bandwidths by the sampling rate, coexisting with the radar system. Set different values of $E_{I}$ to limit the energy distributed over the stopband. We plot the energy spectral density (ESD) of the optimized waveforms in Figure 5, where the reference signal $x_{0}$ is considered as the benchmark.

As we can see, the spectral constraint forces the ESD shape to have a deep notch over the stopbands. The nulls are deeper for smaller $E_{I}$, as less energy is distributed over the frequency band. It indicates that the spectral constraint is effective at achieving spectral compatibility, so that the proposed method allows more than one radiator to work at the same time in a spectrally dense environment.

#### 4.1.5. Time Consumption Comparison

**Figure 6:**
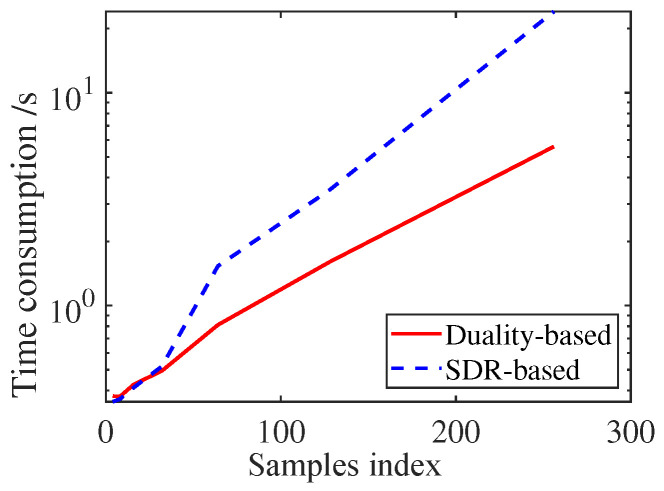
CPU time of both methods to tackle $P_{9}$.

As mentioned before, the SDR technique followed by rank-one decomposition provided in [45] can also solve $P_{9}$, but may cause additional complexity. Herein, the CPU time of the duality-based and SDR-based methods are provided in Figure 6 based on a PC with Inter Core i5 2.4 GHz. As shown, the duality-based method is more computationally efficient than the SDR-based one.

#### 4.1.6. Target Classification

**Figure 7:**
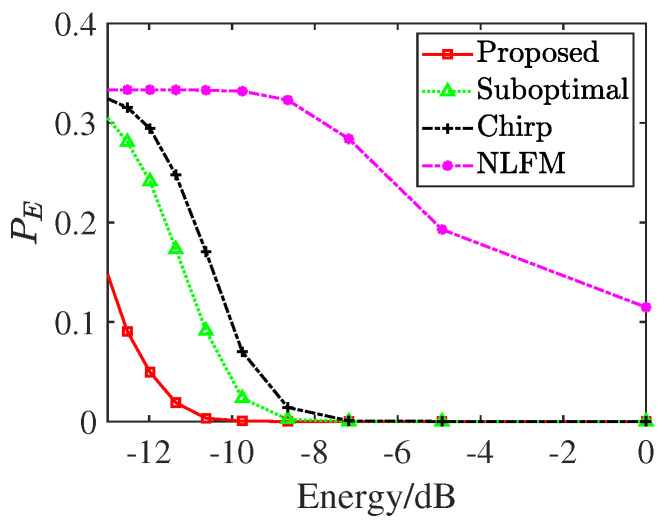
Misclassification probability.

To evaluate the target discrimination capability of the designed waveform, we conducted a classification experiment based on high-resolution range profiles (HRRPs) following the procedure outlined in [48]. Still assume each target appears with equal probability, and the misclassification probability $P_{E}$ under varying waveform energy levels is presented in Figure 7.

Experimental results demonstrate the designed waveform yields lower and higher classification accuracy than benchmarks, particularly at low energy levels. Notably, even at a low normalized energy of −10 dB, the proposed method maintains robust classification accuracy. It further confirms the target detection results presented in Figure 7, highlighting the dual strengths of the proposed approach in both imaging and classification tasks.

### 4.2. Application to SAR

In order to verify whether the resultant waveform can improve the image quality of extended targets, a simple SAR system is simulated. Its parameters are listed in Table 1.

Here, several existing waveforms are considered, including the chirp signal, the NLFM signal [7,49], and the suboptimal waveform [50]. All of them have the same transmit energy, and they illuminate the same scene with an airplane being as the interested target. The area of the airplane is delimited according to its profile, and samples of G(t) are placed into the designated area. Other cells are covered by the samples of background scatters B(t). As the SAR platform advances, the associated SAR echoes are obtained, which are processed by the Range Doppler algorithm to generate the SAR images shown below.

**Figure 8:**
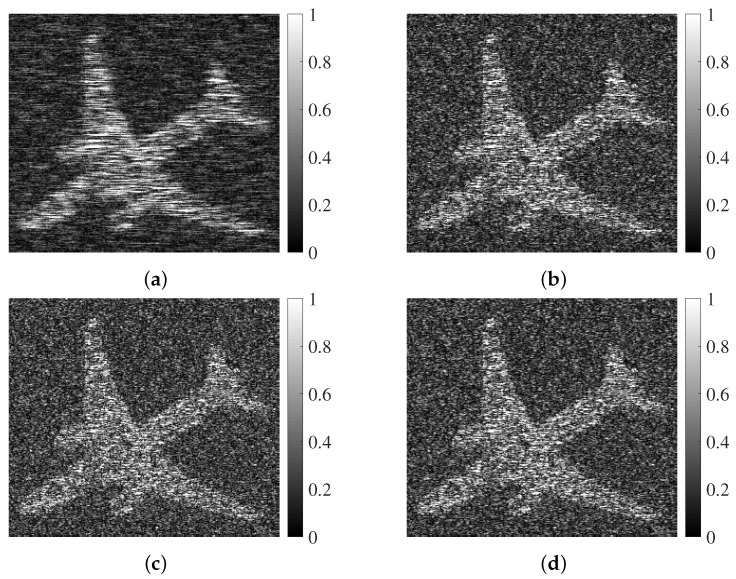
SAR images: (**a**) proposed scheme; (**b**) suboptimal waveform; (**c**) chirp signal; (**d**) NLFM waveform, where the horizontal and vertical axes are the range and azimuth directions in meters, and the SCR of each image is 6.3 dB, 3.8 dB, 2.8 dB, and 3.0 dB, respectively.

**Figure 9:**
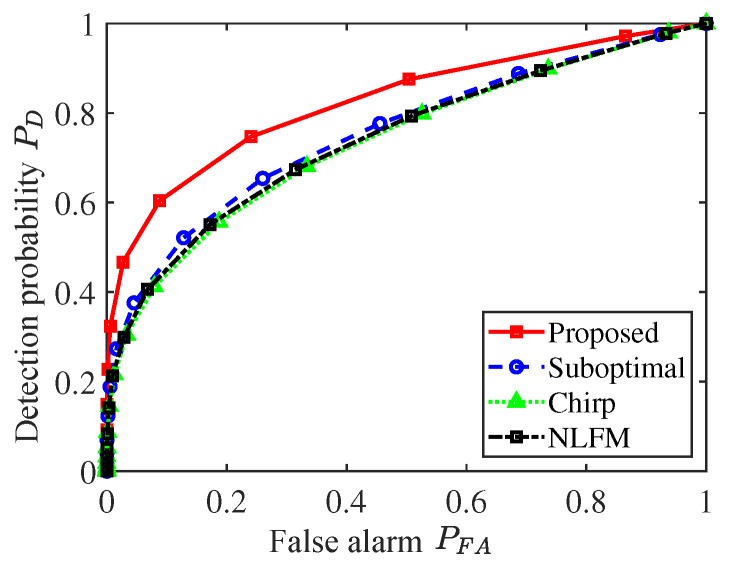
Detection probability and false alarm.

It is obvious that the proposed SAR image achieves the best visual effect for the extended target, compared with all counterparts. As the SCR is improved by the proposed method, a greater contrast between target cells and background scatters are presented. More precisely, the SCR in Figure 8a is 3.5 dB higher than that of the chirp signal. Moreover, since a SAR image with a higher SCR will benefit target discovery performance, an experiment based on the obtained SAR image is conducted to evaluate target detection. Set the threshold of detection to traverse the entire pixel values of SAR image in turn, and the two-value images according to every threshold are generated. Through comparing the ideal case, detection probability $P_{D}$ and false alarm $P_{FA}$ are calculated, as shown in Figure 9.

As expected, a larger SCR leads to higher SAR image quality, further leading to more desired target detection result. In conclusion, the proposed method is effective in highlighting the extended target in a SAR image.

## 5. Conclusions

In this paper, the robust SAR waveform design problem is addressed to improve extended targets’ scattering signature in spectrally crowded environments. It is formulated as a max–min fractional problem, where maximizing the worst-case SCR over the uncertain statistics set is considered as the objective function. Meanwhile, some reasonable constraints, including energy, similarity, and spectrum constraints are introduced to achieve the desired resolution and spectral compatibility. A customized algorithm is developed to tackle the resultant problem. Firstly, the subproblem with the minimizing operator is solved by a closed-form solution. Then, the fractional objective function is coupled by the Dinkelbach’s algorithm, while the Lagrange duality and Schur complement are applied to form the solving procedure performed by iterating a series of SDP problems. In the convergence verification stage, a sufficient condition for global convergence is derived. At the analysis part, the superiority of the proposed design is highlighted by the improvement of the worst-case SCR, while ensuring a high-quality SAR image for the extended targets. Since our method is target-driven, changes in the target to be detected require a redesign of the waveform. In the future, we will further investigate optimized waveforms that are adaptable to multiple target types.

## Figures and Tables

**Table 1:** List of simulation parameters.

Parameter	Symbol	Value
Platform height	$H_{0}$	6 km
Antenna length	*D*	2 m
Effective radar velocity	*v*	150 m/s
Look angle	θ	$30^{\circ }$
Beam squint angle	ϕ	$0^{\circ }$
Center frequency	$f_{0}$	5.3 GHz
Pulse duration	*T*	1 μs
Range Bandwidth	$B_{r}$	100 MHz
Range sampling rate	$F_{r}$	120 MHz
Azimuth sampling rate	$F_{a}$	180 Hz
Number of range lines	$N_{a}$	256
Samples per range line	$N_{r}$	256

## Data Availability

Data are contained within the article.

## Appendix A

### Appendix A.1. Globally Solved P 9 Leads to Global Convergence for P 8

A monotonically increasing sequence ${\left\{f\left(x^{\left(k\right)}\right)\right\}}_{k=1}^{\infty }$ is generated by iterating $P_{9}$. Since f(x) is upper bounded resulting from $x^{H}x\leq E_{x}$, ${\left\{f\left(x^{\left(k\right)}\right)\right\}}_{k=1}^{\infty }$ converges to a limit point, where $f\left(x^{\left(K\right)}\right)=f\left(x^{\left(K-1\right)}\right)$ with a large enough *K*. Let F(x) denote the objective function of $P_{9}$, so that

(A1) $$\begin{array}{ccc} F(x^{\left(K\right)}) & =\alpha \left(x^{\left(K\right)}\right)-\beta \left(x^{\left(K\right)}\right)f\left(x^{\left(K-1\right)}\right)\; & =\alpha \left(x^{\left(K\right)}\right)-\beta \left(x^{\left(K\right)}\right)f\left(x^{\left(K\right)}\right)=0 \end{array}$$

where α(x) and β(x) are the numerator and denominator of f(x), respectively. As $\beta (x^{\left(K\right)})>0$, we have $f\left(x^{\left(K\right)}\right)=\alpha (x^{\left(K\right)})/\beta (x^{\left(K\right)})\;$. Because $P_{8}$ and $P_{9}$ have the same constraints, $x^{\left(K\right)}$ is also a feasible point to $P_{8}$, while

(A2) $$\begin{array}{ccc} f(x^{\left(K\right)}) & =\frac{\alpha (x^{\left(K\right)})}{\beta (x^{\left(K\right)})}\; & \leq f\left(x^{\prime }\right)=\frac{\alpha (x^{\prime })}{\beta (x^{\prime })},\;x^{\prime }\in S\left(P_{8}\right) \end{array}$$

Therefore, $\alpha \left(x^{\prime }\right)-\beta \left(x^{\prime }\right)f\left(x^{\left(K\right)}\right)\geq 0$. As $x^{\prime }$ is also feasible to $P_{9}$, it follows

(A3) $$\alpha \left(x^{\prime }\right)-\beta \left(x^{\prime }\right)f\left(x^{\left(K\right)}\right)\leq \alpha \left(x^{\left(K\right)}\right)-\beta \left(x^{\left(K\right)}\right)f\left(x^{\left(K\right)}\right)=0$$

if $P_{9}$ can be solved globally with $x^{\left(K\right)}\in S\left(P_{9}\right)$ at the *K*-th iteration step. We finally have

(A4) $$\alpha \left(x^{\prime }\right)-\beta \left(x^{\prime }\right)f\left(x^{\left(K\right)}\right)=0$$

which indicates $f\left(x^{\left(K\right)}\right)=\alpha (x^{\prime })/\beta (x^{\prime })\;$. Thus, $P_{8}$ holds with global convergence, if $P_{9}$ is solved globally in each iteration.

### Appendix A.2. Proof of Lemma 1

**Proof.** Let $Y=\left[x;1\right]{\left[x;1\right]}^{H}$, and then

(A5) $$x^{H}A_{i}x+b_{i}^{H}x+x^{H}b_{i}+c_{i}=\mathrm{tr}\left[M_{i}Y\right]$$

where

(A6) $$M_{i}=\left[\begin{array}{cc} A_{i} & b_{i}\\ b_{i}^{H} & c_{i} \end{array}\right]$$

As a result, $P_{10}$ changed to an SDP relaxation problem:

(A7) $$P_{15}\left\{\begin{array}{c} \min_{Y}\mathrm{tr}\left[M_{0}Y\right]\\ \mathrm{s.t.}\;\left\{\begin{array}{c} \mathrm{tr}[M_{i}Y]⪯0,i=1,2,3\\ \mathrm{tr}[\Delta Y]=1\\ Y⪰0 \end{array}\right. \end{array}\right.$$

where $\Delta =\left[\begin{array}{cc} I & 0\\ 0^{H} & 1 \end{array}\right]$, and the rank-one constraint is relaxed. If any $Y\in S(P_{15})$ with $Y=\left[\begin{array}{cc} Y_{11} & y\\ y^{H} & 1 \end{array}\right]$ satisfying $Y_{11}=yy^{H}$, then $V\left(P_{15}\right)=V\left(P_{10}\right)$, and the optimal solution of $P_{10}$ can be obtained through eigen decomposition of Y. The Lagrange dual of $P_{15}$ is

(A8) $$L=\mathrm{tr}\;\;\;\left[\;\;\;M_{0}Y\;\;\;\right]\;\;\;+\sum_{i=1}^{3}\lambda_{i}\mathrm{tr}\;\;\;\left[\;\;\;M_{i}Y\;\;\;\right]\;\;\;-\alpha \left\{\mathrm{tr}\;\;\;[\;\;\;\Delta Y\;\;\;]\;\;\;-1\right\}$$

while the dual function is

(A9) $$\underset{Y⪰0}{\inf}L=\left\{\begin{array}{c} \alpha ,\;M_{0}+\sum_{i=1}^{3}\lambda_{i}M_{i}-\alpha \Delta ⪰0\\ -\infty ,\;\mathrm{otherwise} \end{array}\right.$$

Thus, the dual problem of $P_{15}$ is

(A10) $$P_{16}\left\{\begin{array}{c} \max_{\lambda_{i},\alpha }\alpha \\ \mathrm{s.t.}\left\{\begin{array}{c} M_{0}+\sum_{i=1}^{3}\lambda_{i}M_{i}-\alpha \Delta ⪰0\\ \lambda_{i}\geq 0,\;i=1,2,3 \end{array}\right. \end{array}\right.$$

It is not difficult to find that $P_{16}$ is the same as $P_{13}$. Herein, let (λ,α) and Y be feasible solutions to $P_{16}$ and $P_{15}$, respectively, and then the complementarity slack conditions for $P_{16}$ and $P_{15}$ can be expressed as [51,52]

(A11) $$\begin{array}{c} \mathrm{tr}[\mathrm{HY}]=0\\ \lambda_{i}\mathrm{tr}\left[M_{i}Y\right]=0,\;i=1,2,3 \end{array}$$

where $H=M_{0}+\sum_{i=1}^{3}\lambda_{i}M_{i}-\alpha \Delta$. Assume that at least one of the problems $P_{15}$ and $P_{16}$ is bounded and strictly feasible, and then (λ,α) and Y are optimal if and only if the complementarity slack conditions hold [51]. Now, we prove Lemma 1 is equivalent to determining whether (A11) is satisfied, with $Y^{†}=\left[x^{†};1\right]{\left[x^{†};1\right]}^{H}$. To verify the first part in (A11), we have

(A12) $$\begin{array}{cc} \mathrm{tr}\left[HY^{†}\right] & =\mathrm{tr}\left[A\left(\lambda^{†}\right)x^{†H}x^{†}+b\left(\lambda^{†}\right)x^{†H}\right]+b{\left(\lambda^{†}\right)}^{H}x^{†}+c\left(\lambda^{†}\right)-\alpha^{†}\\ & =-b{\left(\lambda^{†}\right)}^{H}A{\left(\lambda^{†}\right)}^{-1}b\left(\lambda^{†}\right)+c\left(\lambda^{†}\right)-\alpha^{†} \end{array}$$

In Appendix A.3, $\alpha^{†}=c\left(\lambda^{†}\right)-b{\left(\lambda^{†}\right)}^{H}A{\left(\lambda^{†}\right)}^{-1}b\left(\lambda^{†}\right)$ is proved, so $\mathrm{tr}\;[HY^{†}]=0$. Looking back to (9), if $A(\lambda^{†})≻0$, $x^{†}$ solves the unconstrained problem $\min_{x}\;L\left(x,\lambda^{†}\right)$, as $x^{†}$ satisfying the KKT condition $\nabla_{x}L\left(x,\lambda^{†}\right)=0$ [53]. Meanwhile, suppose that $\left(\lambda^{†},\alpha^{†}\right)$ solves the problem

(A13) $$\begin{array}{c} \min_{\lambda_{i}}\;-L\left(x^{†},\lambda \right)\\ \mathrm{s.t.}\;\lambda_{i}\geq 0,\;i=1,2,3 \end{array}$$

Since (A13) is convex, $\left(\lambda^{†},\alpha^{†}\right)$ is an optimal solution, which is also a KKT point. There exist the Lagrange multipliers $\omega_{i}\geq 0,\;i=1,2,3$, such that

(A14) $$\nabla_{\lambda }\left(-L\left(x^{†},\lambda^{†}\right)-\sum_{i=1}^{m}\omega_{i}\lambda_{i}^{†}\right)=0\;\mathrm{and}\;\omega_{i}\lambda_{i}^{†}=0,\;i=1,2,3$$

which further indicates that

(A15) $$\omega_{i}=-\left(x^{†H}A_{i}x^{†}+b_{i}^{H}x^{†}+x^{†H}b_{i}+c_{i}\right),\;i=1,2,3$$

Therefore, combined with (A5), the second part in (A11) $\lambda_{i}^{†}\mathrm{tr}\left[M_{i}Y^{†}\right]=0,\;i=1,2,3$ is confirmed. In conclusion, $Y^{†}$ is optimal to $P_{15}$. Since $Y^{†}$ is also a rank-one solution, $x^{†}$ solves $P_{9}$ through Lagrange duality with zero duality gap. □

### Appendix A.3. Proof of α^†^

**Proof.** For any feasible solution α, we must have

(A16) $$\left[\begin{array}{cc} A(\lambda ) & b(\lambda )\\ b{\left(\lambda \right)}^{H} & c(\lambda )-\alpha \end{array}\right]\underline{≻}0$$

which means that

(A17) $$\alpha \leq c\left(\lambda \right)-b{\left(\lambda \right)}^{H}A{\left(\lambda \right)}^{-1}b\left(\lambda \right)=\tilde{\alpha }$$

Therefore, any feasible α is upper bounded by $\tilde{\alpha }$. Given $\lambda_{i}^{†}\in S\left(P_{13}\right)$, i=1,2,3, to verify $\tilde{\alpha }$ is also feasible, there is

(A18) $$c\left(\lambda^{†}\right)-b{\left(\lambda^{†}\right)}^{H}A{\left(\lambda^{†}\right)}^{-1}b\left(\lambda^{†}\right)-\tilde{\alpha }=0$$

It indicates that $\tilde{\alpha }$ is also a feasible solution for $P_{13}$, and then

(A19) $$\tilde{\alpha }\leq \alpha^{†}\leq \tilde{\alpha }$$

such that

(A20) $$\alpha^{†}=\tilde{\alpha }$$

The proof is completed. □
